# On Sanitary Spelling Schools, with Some Incidental Remarks Concerning Dirty Linen

**Published:** 1884-02

**Authors:** 


### Editorial.

---

### ON SANITARY SPELLING SCHOOLS, WITH SOME INCIDENTAL REMARKS CONCERNING DIRTY LINEN.

The report of the proceedings of the Illinois State Board of Health at its quarterly meeting, October 18–19, 1883, affords an interesting illustration of the difficulties which beset such a body in its efforts to accomplish by forms of law what cannot be properly reached by law.

A considerable portion of the report is occupied by an opinion furnished by the Attorney General of the State, in reply to certain questions raised by the Board concerning prosecutions for a violation of the law of 1877. This official states that this law "can only be maintained under the theory that it is a proper exercise of the police power of the State;" otherwise it "would be an unwarrantable interference with private rights." This "proper exercise of the police power" he further assumes to be "the preservation of the public health from the experiments of quacks and unqualified persons."

As we have shown in a late issue, Mr. Huxley has abundantly proved the folly of any such effort for the preservation of the public health. Mr. Herbert Spencer has also presented the philosophical grounds of such failure, showing that it is inevitable from the very nature of things. Observation of the course of events in our own State during the past six years, brings to light a great amount of evidence that government intervention for the protection of people against quacks simply defeats itself. The impostor merely changes his garb, and pursues his old game without hindrance. He takes pains to arm himself with the proper credentials, signed and sealed by the Board of Health, and to these he can always refer the public as evidence of orthodoxy and competence. In this way hundreds of ignorant and unworthy fellows have been presented to the public as worthy of confidence. The regular physician, who has patiently qualified himself by study and experience, is no better before the law than any unscrupulous humbug who is "recognized" by the Board of Health. Only a few of the stupider sort of quacks run any risk of molestation. An occasional prosecution of these is attempted, creating thus an impression that the Board of Health is doing something to purge the profession, but the practical outcome of such activity has no terrors for the great army of quacks who pursue their game under the protection of the law. This is inevitable, and therefore cannot be a matter for regret. It should, however, be sufficient to open the eyes of those who still believe in the ancient superstition that legislation is the all efficient remedy for the ills of life. As Mr. Huxley has well said, it is much better for people to form the habit of taking care of themselves, than for the State to disarm their suspicions by professing to protect them against quackery. The quack merely learns to keep within the letter of the law, and finds his position established by the endorsement of the State, upon a level of respectability which gives him an advantage against the citizen which he could have obtained in no other way. Our law, therefore, becomes a law, not for the protection of citizens against quackery, but, on the contrary, a law for the protection of quacks against the natural suspicion of cautious citizens. According to the Attorney General of the State, therefore, this law is an "unwarrantable interference with private rights," since its *raison d'etre* has no existence.

Another evidence of the injustice of our present law is furnished by the manner in which it requires the Board of Health to deal with offenders against its provisions. If we are rightly informed, the mode of action is briefly this: The Secretary of the Board of Health decides to make a descent upon some particular offender. He employs detectives to "put up a job" upon the rogue, who generally falls an easy victim to this method of attack. The Secretary examines the evidence thus obtained, and if it seems sufficient, he reports to the full Board at its next meeting, that such or such a physician has given cause for revocation of his license. The Board must as far as possible finish its business in its hours of session. Its non-professional members may be supposed to know nothing, and to care less, about such things. The medical members of the Board are governed by the "courtesy of the court," or by a sense of satisfaction at the thought of making an example of somebody. As a matter of fact, the whole thing is finally left to the Secretary, and whatever he may recommend is tolerably sure to be sanctioned. The criminal then finds himself without any standing in court if he undertakes to continue his practice, because he is then prosecuted, not for unprofessional conduct, but for practicing without a license, so that the justice of his first condemnation cannot be made a question for legal investigation.

Now, the satisfaction with which every right-minded physician contemplates the summary overthrow of an impostor and a charlatan, should not blind him to the enormous injustice of the proceeding just outlined. We hope that there is some mistake in the case, and that the Board of Health may be able to take a position different from that in which it seems to be now placed by the law in its present form. If such be the real mode of dealing with offenders, justice can be obtained from the Board only at its pleasure, for such justice must be secured from a court where the offices of detective, prosecutor, judge and jury are all united in one person. It may be proper to have a public detective set over the medical profession, but he should not be allowed also to act as judge and executioner. All offenders against whom evidence may be secured should be presented by the Board of Health to an independent court of law for adjudication of their offenses. Otherwise, where can be the security that the office of Secretary shall not be employed as a means of injustice and oppression? The ecclesiastical courts which existed in England during the Middle Ages, form the only parallel, concerning which we have any knowledge, to the tribunal of our Board of Health. Organized by the best of men for the most noble and beneficent purposes, those courts became the agents of one of the worst forms of arbitrary tyranny the world had ever known. Such will be the inevitable result if our own Board of Health should be suffered by our apathy to evolve its natural conclusions.

A similar objection lies against many of the measures proposed by the Board of Health in the matter of medical education. The best teachers of medicine will not yield to others in a desire for the progressive elevation of the standard of medical education. But there must be evidence that the line of action marked out by legislation is judicious and likely to be successful, before there can be a cheerful acceptance of everything that is forced upon the medical colleges, with or without their consent. It is very evident that there are two sides to this whole question of medical education. The experience of our English brethren shows how difficult and delicate is the proper adjustment of the matter. One would, therefore, suppose that before initiating any new de-departure in this State, our Board of Health would at least have consulted with the members of the medical corporations most likely to be affected by their action. In Great Britain, when any legislation affecting medical education or the interests of the medical profession, is undertaken, the whole matter is first subjected to the most searching inquiry by the representative members of the bodies interested. It is ardently debated by all the medical journals throughout the Kingdom; the "fierce light of parliamentary discussion" is poured like a flood upon it; every effort is made to secure the rights and privileges of all parties concerned. But in this State nothing of the kind is attempted. A little knot of irresponsible gentlemen, some of them physicians and some of them not—all with little or no experience in teaching scientific medicine—lay their heads privately together, and, without asking advice, or even an expression of opinion from any one, they proceed to dictate to the medical colleges in matters affecting some of their most vital interests. In this way they have seized upon a vantage ground whence they can inflict irreperable injury upon any institution which may fall under the ban of their displeasure. Their reports are garnished with the names of colleges which are "not recognized by the Board"—names which are paraded with evident gusto, very much as an Indian brave points to the scalp locks dangling at his belt; and we all admire and applaud the good work of "purging the profession."

But a little investigation reveals the fact that this action is entirely arbitrary, and that no medical college, however venerable or excellent its record, is safe against any assault which the Board of Health may choose to bring against it. Of course it is very easy to insist that there is no danger of any harsh or unjust treatment from the Board, but this does not remove the fact that there exists no legal barrier between the Board and any medical college whatsoever. A most conspicuous example of the consequences of this fact is exhibited by the indifference of the Board to all conference with the medical colleges of the State in the matter of medical education.

An excellent illustration of the unwarrantable character of certain of the exactions made by the Board of Health is contained in the admission by the Secretary concerning their effects upon medical colleges which have adopted a three years graded course. The only way out of the difficulty, which the Secretary would propose in a case which actually presented itself, consisted in a virtual evasion of the rules adopted by the Board. As a matter of fact, if the Board should attempt to enforce their own rules it would soon result in the practical crippling of any school within the State which might adopt the three years' graded course. How can this be reconciled with a judicious zeal for the elevation of the standard of medical education ?

Again, in the matter of preliminary qualifications for admission to the medical colleges of the State, it is difficult to see how the action of the Board is likely to accomplish any good result. There is no unanimity of opinion regarding the value of such preliminary examinations as are required. We have already, in our last issue, quoted the opinion of Prof. Huxley; and the utterances of such a man as Prof. Knapp (see his recent inaugural address, published in the *Medical Record*) are of the highest importance in this connection. The whole matter is complicated by so many conflicting interests, growing out of the independence of State governments, rendering impossible any concerted action throughout the Union, that our State Board should undertake nothing without the fullest consultation and most cordial coöperation with all the colleges in the State. On the contrary, the Board, by its arbitrary action, is simply hindering the natural and healthy development of the medical colleges, and, under the plea of raising the standard of education, is only driving timid students out of the State, into inferior schools, where they cannot secure anything like a metropolitan education. The fledgling medical colleges upon the frontier can well afford to pay our Board liberally for this kind of discrimination in their favor.

The insignificance of the matters which chiefly occupy the Board is further shown by the prominence given to the subject of spelling. Nearly three pages of this brief report are filled with samples of letters received from would-be medical students and frontier physicians. Previous reports have been filled with similar specimens. We would suggest that this subject has now been sufficiently treated by the Board, unless they wish to be considered a spelling school. Of course, there can be no harm in exhibiting such letters to the different members in private sessions of the Board. College Faculties have been known to amuse themselves for a few moments with similar effusions from a newly plucked candidate ; but that is something very different from the parade of such errors before an unprofessional public. The Board should remember that their correspondence is chiefly with the riff-raff of the medical profession, and with such young students as do not know any better way of obtaining information. It is, therefore, to be expected that their correspondence will be unusually fertile in examples of bad spelling and feeble grammar. Every one who is acquainted with the young men who compose the classes in our respectable medical schools, knows that such errors are exceedingly rare among them at the present time, whatever may have been true when the medical members of the Illinois State Board of Health were students. It is, therefore, a very injudicious thing to make so much of a little matter as has been done in this and in preceding reports. They are widely circulated, and are copied into the newspapers, giving to the general public the impression that physicians and medical students are, as a rule, exceedingly illiterate. For example, a late number of the *London Medical Press and Circular* made a quotation in its columns from the reports of the Illinois Board on the subject of spelling; and if, as a result of the publicity thus acquired, a large proportion of the practitioners in Great Britain do not conclude that most American physicians do not know how to spell, they will certainly exhibit more judgment than they have usually done in forming opinions as to this country and its inhabitants.

In this way public opinion is led very far astray, and the medical profession suffers correspondingly in the popular estimation. Dirty linen should be quietly washed at home, instead of being flaunted in the face of every passer-by. "It is an ill bird that fouls its own nest," is an old saw that should not be allowed to sink into oblivion.

We remark, in conclusion, that the periodical reports of the Board produce the impression that very little work of a strictly sanitary character is occupying its time. Mousing after irregular practitioners, "steering" medical students, nursing their pet medical colleges, and stuffing the newspaper reporters with Falstaffian narratives of their combats with the quacks—these seem to be the favorite pursuits which arouse the enthusiasm of the authors of these reports. The question of the particular value of such work to the general public must inevitably at length arise for discussion. The consequences of such discussion will not be averted by anything short of a reformation of our present State law.

